# Epitranscriptomic control of cancer immunity and therapy resistance

**DOI:** 10.3389/fimmu.2025.1706557

**Published:** 2025-11-07

**Authors:** Xingsen Zhao, Suhua Guan

**Affiliations:** 1 Institute of Biotechnology, Xianghu Laboratory, Hangzhou, Zhejiang, China; 2 Institute of Biomanufacturing Research, Xianghu Laboratory, Hangzhou, Zhejiang, China

**Keywords:** epitranscriptomics, RNA modifications, tumor microenvironment, immunotherapy, M6A, m5C, m1A, A-to-I editing

## Abstract

Epitranscriptomics, the study of dynamic chemical modifications on RNA mediated by “writers,” “erasers,” and “readers,” has emerged as a pivotal discipline in elucidating the intricate interplay between cancer and immune regulation. These reversible modifications (e.g. m^6^A, m^5^C, Ψ) govern RNA metabolism, stability, and translation, thereby exerting spatiotemporal control over immune cell differentiation, activation, and function. Dysregulation of RNA-modifying proteins disrupts immune surveillance, enhances tumor cell survival under stress, and promotes chemoradiotherapy resistance by altering RNA splicing, translation, and stress adaptation pathways. This review summarized the recent progress in the regulatory mechanisms profoundly influencing the tumor microenvironment (TME), modulating immune checkpoints, antigen presentation pathways, and the activity of immune cells. Furthermore, we discussed the therapeutic strategies and challenges in targeting epitranscriptomic regulators and epitranscriptomic editing technologies to enhance anti-tumor immune responses and overcome therapeutic resistance.

## 1 Introduction

**Figure 1:**
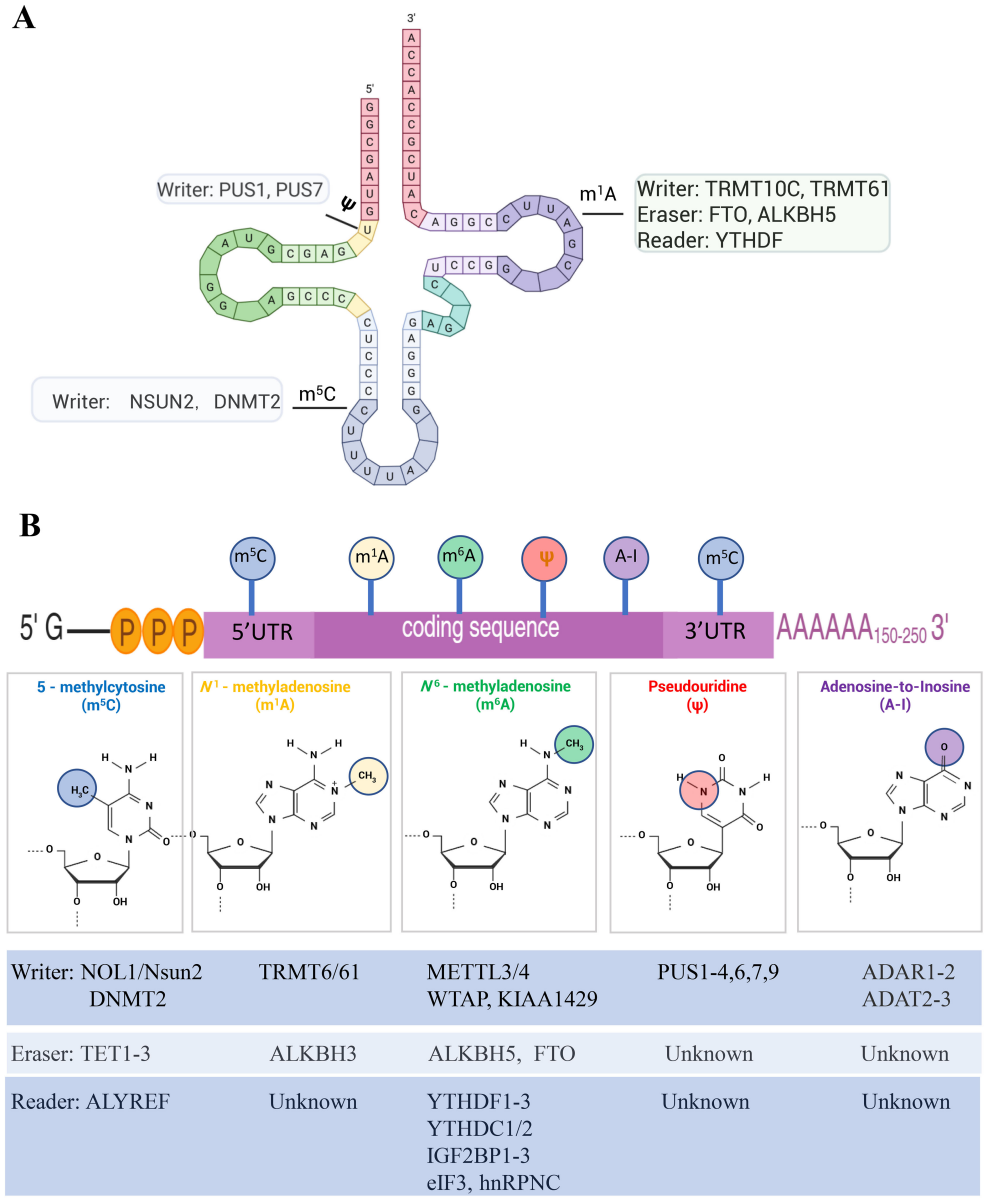
RNA modification on different RNA moleculars. **(A)** On tRNA moleculars, m^1^A is catalyzed by TRMT61B and TRMT10C. Erasers of m^1^A include the ALKBH family and FTO. YTHDF can recognize m^1^A modifications. m^5^C can be catalyzed by NSUN5 and may cause read-through of stop codons on rRNA. **(B)** RNA modifications on mRNA molecular. m^5^C: methylation occurs at the 5th position of cytosine residues. m^1^A: modification occurs at the first N atom of the adenine base. It is associated with processes such as RNA stability, stress-induced granulation, and trophoblast invasion. m^6^A: methylation of adenosine at the N6 position, widely found in the exonic and 3’-end untranslated regions of all types of RNAs (e.g., mRNAs and non-coding RNAs). Ψ: Pseudouridine is a natural structural analogue of uracil nucleosides (U), except that in its ring structure a hydrogen bond is formed between the carbon 1 and nitrogen 1 positions. A-to-I RNA editing:mediated by ADAR (adenosine deaminase acting on RNA) proteins. Because I is recognized as G, A-to-I RNA editing spatiotemporally and spatially specific increases transcriptome and proteome diversity without altering the genome sequence.

Cancer cells deploy an arsenal of immune-evasion strategies to persist within the host, from downregulating antigen presentation to exploiting checkpoint pathways. In recent years, epitranscriptomics has emerged as a pivotal modulator of the cancer–immunity axis. Epitranscriptomic marks refer to chemical modifications on RNA molecules that fine-tune their processing, stability, localization, and translation without altering the underlying sequence. These marks are installed by “writers” (e.g., methyltransferases such as METTL3/METTL14 for N^6^-methyladenosine, m^6^A), removed by “erasers” (demethylases like FTO and ALKBH5), and interpreted by “readers” (binding proteins such as the YTHDF/YTHDC family) (1). Collectively, this dynamic “writer–eraser–reader” network orchestrates gene expression programs that govern both tumor cell biology and immune cell function (2, 3) (**Table 1**; **Figure 1**).

**Table 1:** Enzymes in mRNA modifications and their Key roles in cancer & immunity.

Enzyme (complex)	Class	Key roles in cancer & immunity
METTL3/METTL14	Writer (m^6^A)	Stability/translation of DDR, ferroptosis, and niche genes; broad tumor/immune roles
METTL16	Writer (m^6^A, substrate-specific)	SAM-sensing via MAT2A; transcript-selective control
FTO	Eraser (m^6^A/m^6^Am)	HR/DSB repair competence; RT resistance context
ALKBH5	Eraser (m^6^A)	GSC radioresistance; stemness & chemotolerance
YTHDF1	Reader (m^6^A)	DC cathepsins increase STING degradation; IFN-I decrease
YTHDF2	Reader (m^6^A)	Myeloid suppressive program maintenance
IGF2BP1/2/3	Reader (m^6^A)	mRNA stabilization (ABCB1, YAP1, others) inducing drug resistance
NSUN6	Writer (m^5^C)	Stabilizes NDRG1; RT resistance in cervical models
NAT10	Writer (ac^4^C)	Stabilizes DDX41, ZNF746; dacarbazine resistance
ADAR1	Editor (A-I)	MDA5-IFN dampening; PCNA/RAD18 tolerance

m^6^A methylation is the most abundant internal modification in eukaryotic mRNA and has been extensively linked to immune regulation in cancer. Transcriptome-wide mapping studies reveal that METTL3 and METTL14 deposit m^6^A on transcripts encoding key costimulatory and coinhibitory molecules—including CD70, CD80, and TIGIT—thereby modulating their mRNA stability and cell-surface expression across diverse solid tumors. Such regulation directly impacts T-cell activation thresholds and tumor immune escape mechanisms (4). Equally, the m^6^A “eraser” ALKBH5 controls the demethylation of PD-L1 mRNA; loss of ALKBH5 destabilizes PD-L1 transcripts via enhanced YTHDF2 binding, increasing tumor vulnerability to T-cell–mediated lysis and potentiating responses to PD-1 blockade in preclinical glioblastoma models (5). This dual influence on both costimulatory ligands and inhibitory checkpoints underscores m^6^A modification as a master regulator of the tumor–immune interface.

Beyond m^6^A, a growing body of work highlights the immunomodulatory roles of additional RNA modifications. 5-Methylcytosine (m^5^C), installed by NSUN2 and DNMT2, tunes the translation efficiency of cytokine and chemokine transcripts, thereby shaping the immune-cell recruitment landscape within tumors. Pan-cancer analyses demonstrate that elevated m^5^C levels correlate with enhanced expression of CXCL10 and CCL5, improving CD8^+^ T-cell infiltration and sensitizing otherwise “cold” tumors to checkpoint blockade (6). In parallel, N^1^-methyladenosine (m^1^A) marks have been mapped on transcripts encoding interferon-stimulated genes, where they regulate ribosome pausing and translation initiation, fine-tuning the amplitude and duration of type I interferon responses during anti-tumor immunity (7).

Another modification gaining attention is pseudouridine (Ψ), which alters RNA secondary structure and base-pairing properties. Nucleoside-modified mRNA (e.g., Ψ or m¹Ψ) can increase translation and dampen innate sensing (8, 9), and such platforms elicit robust CD8^+^ T-cell responses in vaccine settings (10). Moreover, site-specific pseudouridine within coding sequences can alter translation kinetics and decoding, potentially implying complex effects on the immunopeptidome (11).

Adenosine-to-inosine (A-to-I) RNA editing, mediated by ADAR1, represents a distinct epitranscriptomic mechanism with profound immune implications. By “self-marking” endogenous double-stranded RNAs, ADAR1 prevents aberrant activation of cytosolic RNA sensors such as MDA5, thereby limiting tonic type I interferon signaling that would otherwise trigger tumor cell immunogenicity. Genetic or pharmacologic inhibition of ADAR1 in preclinical models restores interferon production, heightens natural killer (NK) and T-cell infiltration, and synergizes with anti-PD-1 therapy to drive durable tumor regression (12). These data establish ADAR1 as a gatekeeper of RNA-sensing pathways and a promising target for combination immunotherapy.

Technological advances have been critical in unveiling the complex epitranscriptomic landscapes within the tumor microenvironment. Direct RNA sequencing (DRS) platforms, especially those based on nanopore technology, now permit simultaneous detection of multiple modifications—m^6^A, m^5^C, Ψ, and more—at single-molecule resolution. Recent methodological improvements allow unbiased mapping of >10 distinct marks in patient-derived tumor biopsies, revealing cancer-type–specific “modification signatures” that correlate with immune-cell infiltration patterns and clinical outcomes (13, 14). Such comprehensive profiling is accelerating the discovery of epitranscriptomic biomarkers predictive of immunotherapy response.

Given the central roles of RNA modifiers in shaping anti-tumor immunity, small-molecule inhibitors targeting these enzymes are rapidly advancing. The FTO inhibitor FB23–2 increases global m^6^A levels, suppresses leukemic stem-cell self-renewal, and enhances the expression of pro-immune cytokines in acute myeloid leukemia models; when combined with ibrutinib or checkpoint blockade, FB23–2 augments cytotoxic T-cell activity and improves survival (15). Similarly, ALKBH5 inhibitors—such as MV1035 analogs—have been shown to reduce PD-L1 expression and rescue anti-tumor immunity in glioblastoma allografts, highlighting the potential of “eraser” blockade to overcome immune escape (5, 16). Early-phase clinical trials of FTO and ALKBH5 inhibitors, both as monotherapies and in combination with PD-1/PD-L1 antibodies, are now in planning stages, underscoring the translational promise of epitranscriptomic-based immunomodulation.

In this review, we first examine the roles of m^6^A methylation “writers,” “erasers,” and “readers” in modulating co-stimulatory molecules and immune checkpoints within the tumor microenvironment; next, we explore the diverse functions of non-m^6^A modifications—m^5^C, m¹A, and pseudouridine—in tuning cytokine translation, interferon-stimulated gene expression, and antigen presentation; we then analyze ADAR1-mediated A-to-I editing as a critical regulator of RNA sensing and type I interferon signaling, including its therapeutic potential in combination with PD-1 blockade; following this, we review cutting-edge direct RNA sequencing and multi-omic approaches for mapping epitranscriptomic landscapes in clinical biopsies, highlighting both technical breakthroughs and standardization challenges; and finally, we discuss emerging small-molecule inhibitors of FTO and ALKBH5, evaluating their pharmacology, safety considerations, and strategies for integrating epitranscriptomic modulation with existing immunotherapies.

## 2 Core mechanisms of epitranscriptomic regulation

Epitranscriptomic modifications are installed, removed, and interpreted by a sophisticated network of proteins, collectively referred to as the “writers,” “erasers,” and “readers.” This regulatory system provides dynamic and precise control over RNA fate and function.

### 2.1 Molecular basis of RNA modifications

#### 2.1.1 Common types of RNA modifications (m^6^A, m^5^C, Ψ, etc.) and their chemical properties

RNA carries more than 150 distinct chemical marks that go well beyond its four-letter code, and three in particular—N^6^-methyladenosine (m^6^A), 5-methylcytosine (m^5^C), and pseudouridine (Ψ)—have taken center stage because of their outsized impact on everything from RNA folding to immune signaling.

##### 2.1.1.1 m^6^A: the versatile methyl “switch”

At the N^6^ position of adenosine, the methyl group projects into the RNA major groove and typically modestly weakens an A–U pair rather than fully disrupting it, with context-dependent duplex destabilization of roughly 0.5–1.7 kcal·mol^-1^ and accompanying conformational biasing of the base pair (17). In structured elements, this subtle energetic tilt can remodel local secondary structure—the classic “m^6^A switch”—to expose otherwise occluded motifs and enhance binding of indirect, structure-sensitive readers such as HNRNPC and HNRNPG, thereby influencing splicing and gene expression (18). Notably, a structural “switch” is not a prerequisite for regulation. YTH-family readers can recognize m^6^A and act without extensive RNA unfolding, and recent overviews suggest the YTHDF paralogs often function redundantly to drive mRNA decay—while the details remain context-dependent and influenced by low-complexity regions and condensate formation, which are still being actively debated (19). Beyond mRNAs, m^6^A on U6 snRNA adjusts spliceosome architecture and splice-site selection, emphasizing that m^6^A’s structural influence reaches the core splicing machinery (20). Finally, systems-level studies of Xist indicate that m^6^A deposition interfaces with NEXT-mediated turnover to shape X-inactivation dynamics, underscoring a broader role for m^6^A in controlling lncRNA stability and the timing of dosage compensation (21).

YTH-domain readers (e.g., YTHDF1–3) harbor a conserved aromatic cage that specifically binds the methyl group with high affinity (K_D ≈ 100 nM), recruiting effectors that drive either mRNA decay or translation enhancement (22–24) (**Figure 1B**). For example, m^6^A directs transcripts for rapid turnover via YTHDF2 or promotes efficient translation via YTHDF1 recruitment (24). In immune contexts, dynamic m^6^A deposition on cytokine-receptor and checkpoint-ligand mRNAs calibrates T-cell activation thresholds, while in tumors altered m^6^A landscapes on PD-L1 and CD80 transcripts modulate immune evasion and sensitivity to checkpoint blockade (25, 26). High-resolution m^6^A mapping using photo-crosslinking–assisted techniques (PA-m^6^A-seq) now achieves single-nucleotide resolution, revealing context-specific m^6^A sites that correlate with patient responses to PD-1 inhibitors in melanoma (27).

##### 2.1.1.2 m^5^C: the stability booster

The m^5^C modification occurs in both coding and non-coding RNAs, and plays essential roles in RNA stability and translation efficiency. The methyl group added at the C^5^ position of cytosine’s pyrimidine ring is catalyzed primarily by methyltransferases such as NSUN2 and DNMT2. The export adaptor ALYREF selectively binds m^5^C-marked transcripts, coupling methylation to nuclear export (28).

Chemically, m^5^C increases the base hydrophobicity without disrupting the Watson–Crick edge. The presence of m^5^C enhances base-stacking interactions and backbone rigidity, subtly increasing duplex stability by ~0.5 kcal/mol per modification (29). This reinforcement favors higher-order structures and can enhance the assembly of ribonucleoprotein complexes. Coversely, aberrant m^5^C levels have been linked to various diseases, including cancer, where they may contribute to tumorigenesis by altering the expression of oncogenes and tumor suppressor genes (30).

##### 2.1.1.3 N¹-Methyladenosine

N¹-methyladenosine (m¹A) carries a positive charge at physiological pH because methylation at N¹ quaternizes the ring nitrogen. This modification blocks the Watson–Crick edge and thereby disrupts normal A–U base pairing; Moreover, A-form RNA also disfavors the compensatory A–U Hoogsteen geometry, so local duplexes tend to destabilize rather than “switch” (31). Consistent with these physical constraints, m¹A in the 5′ UTR or near start codons can remodel secondary structure and local electrostatics to alter 40S scanning and start-site selection, yielding context-dependent outcomes—enhanced initiation on some transcripts and scanning impediments on others (32). At the disease level, dysregulated m¹A machinery is linked to malignant phenotypes: overexpression of the TRMT6/TRMT61A writer complex correlates with poor prognosis and supports proliferation and stress tolerance in multiple models (glioma, bladder cancer, hepatocellular carcinoma), while the m¹A demethylase ALKBH3 promotes cancer cell growth, invasion, and—in several reports—therapy resistance (33).

##### 2.1.1.4 Pseudouridine: the RNA shape-shifter

Pseudouridine (Ψ) is the isomerization of uridine relocates the glycosidic bond from N¹ to C^5^ of the uracil ring, introducing an extra hydrogen-bond donor (N¹–H) and altering sugar–base geometry. The additional hydrogen donor and modified torsion angle strengthen base stacking and increase local thermal stability (34). These structural changes favor more rigid helices and sharper bends in single-stranded regions. Pseudouridine synthases (e.g., PUS1, PUS7) install Ψ across rRNAs, tRNAs, snRNAs, and mRNAs. Nanopore direct RNA sequencing now distinguishes Ψ modifications by altered ionic current signatures, enabling single-molecule detection of pseudouridylation patterns in tumor biopsies and correlating specific Ψ sites with immune infiltrate density (35). Monroe et al. used both biochemical and computational approaches to show that Ψ (and m¹Ψ) substitutions in coding mRNAs alter the energetics of codon–anticodon pairing. These modifications change how the ribosome samples near-cognate tRNAs in the A-site, reducing miscoding and thus have the potential to enhance both the speed and accuracy of protein synthesis from modified mRNAs (34). Closely related work on conserved pseudouridines in helix 69 of the rRNA shows that removing these Ψ residues leads to increased frameshifting and stop-codon read-through, underscoring the broader principle that pseudouridylation supports translational fidelity (36).

In summary, these RNA modifications—methyl groups or isomerized bases—uniquely reshape hydrogen bonding, base stacking, and charge, thereby directing RNA structure and interactions. By decoding these physicochemical “marks,” cells regulate mRNA processing, translation, and stability in real-time, enabling both immune cells and cancer cells to adapt swiftly to microenvironmental cues. Understanding these chemical underpinnings is crucial for harnessing epitranscriptomic pathways as novel cancer immunotherapy targets.

#### 2.1.2 RNA modification “writers,” “erasers,” and “readers” proteins and their functions

The intricate regulation of RNA modifications, particularly N^6^-methyladenosine (m^6^A), is mediated by three key classes of proteins: writers, erasers, and readers. The m^6^A “writer” complex extends beyond the core METTL3–METTL14 heterodimer to include several accessory factors that fine-tune substrate specificity and activity. METTL3 and METTL14, together with WTAP, VIRMA (KIAA1429), RBM15/15B, and ZC3H13, form the methyltransferase complex (MTC) that installs m^6^A on consensus RRACH motifs across coding and non-coding RNAs (37–39). METTL16 has emerged as an m^6^A writer with an independent substrate spectrum, targeting U6 snRNA and the 3′-UTR of MAT2A transcripts to regulate S-adenosylmethionine homeostasis (39). More recently, the VCR (vertebrate‐conserved region) of METTL16 is essential for U6 snRNA recognition and catalytic efficiency, underscoring METTL16’s independent substrate specificity beyond METTL3/METTL14 machinery (40). Dysregulation of these writers reshapes the tumor microenvironment (TME): for example, METTL3 overexpression in tumor cells promotes myeloid-derived suppressor cell (MDSC) accumulation via the BHLHE41–CXCL1/CXCR2 axis, dampening CD8^+^ T-cell responses (41).

Counterbalancing methylation, the “erasers” FTO and ALKBH5 dynamically remove m^6^A marks to modulate RNA fate. In several solid tumors, ALKBH5 regulates MDSC infiltration and T-cell priming, and its inhibition enhances checkpoint blockade efficacy (42). FTO-mediated demethylation of key transcripts (e.g., PD-L1 mRNA) has been implicated in tumor immune evasion and resistance to anti-PD-1 therapy (43–46). Beyond m^6^A, emerging evidence identifies ALKBH3 as a demethylase for m^1^A and m^3^C in tRNAs, influencing cellular stress responses; its role in cancer immunity remains to be fully elucidated but represents a promising frontier (32, 47–49).

“Reader” proteins decode m^6^A marks to effect downstream outcomes. The YTH domain family—including nuclear YTHDC1 and YTHDC2, and cytoplasmic YTHDF1–3—bind m^6^A to direct splicing, export, translation, or decay. YTHDF2 promotes degradation of m^6^A-modified transcripts, whereas YTHDF1 enhances their translation (22, 50, 51). In the immune context, YTHDF1 loss in dendritic cells boosts cross-presentation of tumor antigens and synergizes with anti-PD-1 treatment (52–54). In NK cells, YTHDF2 enhances antitumor and antiviral immunity by promoting the degradation—rather than stabilization—of m^6^A-modified transcripts (notably *Tardbp* and *Mdm2*), thereby sustaining IL-15/STAT5-dependent survival, maturation, and effector functions (55). Beyond YTH proteins, the IGF2BP family (IGF2BP1–3) binds m^6^A to stabilize oncogenic mRNAs such as PD-L1, enhancing immune escape in colorectal and breast cancers (56–58). Heterogeneous nuclear ribonucleoproteins (HNRNPA2B1, HNRNPC) and eIF3 also serve as readers, linking m^6^A to alternative splicing and cap-independent translation under stress (59–61).

The functional interplay among writers, erasers, and readers is exquisitely sensitive to cellular state and external cues. In response to viral mimicry, METTL3-mediated m^6^A on RIG-I transcripts modulates innate antiviral signaling and may influence tumor-associated inflammation (62, 63). Hypoxia and nutrient stress in the TME reprogram writer and eraser expression, altering mRNA stability and translation to favor either tumor survival or immune activation (64, 65). High-throughput and proximity proteomics have begun to map the protein neighborhoods of RNA granules with much finer granularity, revealing cooperating regulators beyond the canonical YTH family. Notably, FMRP (FMR1) directly binds YTHDF1 and gates its condensation with ribosomal components; stimulus-induced phosphorylation of FMRP releases YTHDF1 to potentiate translation of its mRNA targets. Parallel interactomic and mechanistic work places PRRC2A/B/C within stress-granule–linked initiation assemblies, where these factors engage pre-initiation complexes and promote leaky scanning; PRRC2C, in particular, is required for efficient stress-granule formation. Taken together with the requirement for YTHDF proteins themselves in stress-granule assembly, these data point to a cooperative, multilayered epitranscriptomic control of RNA-granule dynamics under stress (66–68).

In adoptive cell therapy, targeted modulation of writers is also emerging as a lever to reshape cell fate. Recent preclinical work shows that depleting METTL16 in CAR-T cells increases TCF-1 (TCF7) by reducing its m^6^A -dependent down-regulation, thereby fostering a TCF-1^+^ precursor-exhausted (TPEX) program associated with superior persistence and antitumor activity. These findings argue that tuning m^6^A installation can recalibrate CAR-T differentiation trajectories and durability *in vivo* (69).

The dynamic balance of m^6^A writers, erasers, and readers orchestrates RNA metabolism in both cancer and immune cells. Therapeutic strategies that inhibit METTL3 (small molecules in preclinical development), ALKBH5, or YTHDF1 reshape the TME to enhance antigen presentation and effector T-cell function. As our mechanistic understanding deepens—through integrated omics and refined RNA-binding assays—novel epitranscriptomic targets will undoubtedly emerge, offering promising avenues for precision immunotherapy.

#### 2.1.3 Dynamic regulation of RNA modifications and their impact on RNA metabolism

RNA modifications are written, read, and erased in a highly dynamic, context-dependent manner that tunes RNA fate across the entire life cycle of the transcript. Among them, m^6^A remains the best-characterized example of a reversible mark, and its effects depend on when and where it is deposited and which reader circuits are engaged. Recent syntheses emphasize that m^6^A sets not only translation and decay rates but also couples nuclear processing to cytoplasmic turnover—allowing cells to reprogram gene expression on short timescales during development and stress (70).

Mechanistically, m^6^A is frequently deposited co-transcriptionally, where it can influence termination and genome integrity. In mammalian cells, the helicase DDX21 recruits the METTL3/14 complex to R-loops, promoting proper termination and safeguarding genome stability—direct evidence that functionally links m^6^A writing to transcriptional mechanics (71). Once marked, nuclear readers help determine the first “branch points” of RNA metabolism. YTHDC1 promotes selective nuclear export of m^6^A -tagged transcripts through SRSF3–NXF1 and related adaptors, and YTHDC1-driven condensates can gate export under disease conditions—underscoring the idea that nuclear m^6^A recognition programs downstream cytoplasmic fates (72).

In the cytoplasm, YTHDF paralogs generally bias marked mRNAs toward CCR4–NOT-coupled deadenylation and decay—a “unified model” supported by loss-of-function genetics and quantitative transcriptomics. At the same time, division of labor and context specificity exist across DF proteins and low-complexity domains (70, 73). A complementary circuit is provided by IGF2BP1-3, which stabilize many m^6^A-bearing mRNAs. For example, IGF2BP3 preserves NOTCH3 transcripts by suppressing CCR4–NOT-mediated deadenylation in an m^6^A-dependent manner, illustrating how distinct readers can route the same chemical mark to opposing outcomes (74).

How m^6^A reshapes RNA partitioning during stress has been clarified by recent single-molecule and fractionation studies. On the one hand, limited or context-dependent roles for m^6^A in targeting bulk mRNAs to stress granules (SGs) have been documented in Mettl3-deficient settings (75). On the other hand, long mRNAs that are rich in m^6^A show length-dependent, DF-assisted enrichment in SGs, and YTHDF2–G3BP1 interactions can modulate SG stability—together suggesting that stress-induced sorting depends on transcript features, m^6^A density, and reader availability rather than a single deterministic rule (76, 77).

Early reports proposed that 5′UTR m^6^A broadly promotes cap-independent initiation. A rigorous 2024 study revisited these claims and found that 5′UTR m^6^A does not generally enhance initiation, refocusing attention on coding-region and 3′UTR marks, reader engagement, and coupling to decay as principal levers of output (78).

Other prevalent marks add further layers. m^5^C, written by NSUN2 and read by ALYREF, can enhance the nuclear export, stability, and translation of target mRNAs in cancer models; ALYREF also cooperates with ELAVL1 to amplify m^5^C-dependent export and oncogenic programs (79, 80). Emerging work in immuno-oncology links the NSUN2–ALYREF axis to PD-L1 upregulation and immune evasion, highlighting RNA-modification circuitry as a tractable point of intervention in the tumor–immune dialogue (81). For pseudouridine (Ψ), 2024 BACS chemistry delivers absolute, base-resolution maps and stoichiometry—an enabling technology that is already revealing site-specific Ψ regulation across coding and noncoding RNAs and will facilitate more causal tests of Ψ-dependent translation phenotypes (82).

Technical advances now let us watch these dynamics at relevant scales. picoMeRIP-seq profiles m^6^A from picogram-level input and single embryos; sn-m^6^A-CUT&Tag co-profiles nuclear m^6^A marks with transcriptomes in single nuclei—tools that concretely shift the field from static snapshots to cell-state-resolved, time-aware maps of RNA modification and fate (83, 84).

Across cancers, altered abundance or localization of writers, erasers, and readers reshapes splicing, translation, and damage responses. In the tumor immune microenvironment, multiple reviews now integrate mechanistic and translational data, linking m^6^A/m^5^C circuitry to antigen presentation, interferon signaling, checkpoint regulation, and resistance to immunotherapy—thereby motivating therapeutic targeting of readers/writers and axis-level rewiring strategies (47).

Evidence for m^6^A in immune regulation is the most robust, with independent studies consistently showing its impact on T-cell activation thresholds and antigen presentation. By contrast, roles for m^5^C and Ψ in immune cells remain underexplored, with most findings being correlative. Further cell-type–specific *in vivo* models are needed to validate their immune functions.

### 2.2 The role of RNA modifications in immune cell differentiation and function

RNA modifications shape how immune cells differentiate, traffic, and execute effector programs inside tumors. While tumor-intrinsic epitranscriptomic changes remodel antigenicity and cytokine landscapes, immune cell-intrinsic marks such as m^6^A and A-to-I editing directly tune lineage decisions, metabolic fitness, and cytotoxic potential.

#### 2.2.1 CD8^+^ T cells and Tregs

Multiple groups have demonstrated that m^6^A machinery constrains or reinforces T-cell effector programs in tumors, but the effects are context-dependent. In murine melanoma and colon cancer models, pharmacologic or genetic inhibition of the writer METTL3 enhanced tumor control by sustaining CD8^+^ T-cell cytotoxicity and reducing exhaustion-associated features, thereby improving responses to anti-PD-1; single-cell RNA-seq also showed expansion of activated, less-exhausted CD8^+^ states under METTL3 inhibition (85). Mechanistically orthogonal studies links the m^6^A reader YTHDF2 to CD8^+^ T-cell state transitions: perturbing YTHDF2 rewires translational control programs associated with antitumor activity and responsiveness to checkpoint blockade (86). By contrast, Treg-specific epitranscriptomic programs tend to dampen antitumor immunity. Conditional deletion of Ythdf2 in Foxp3^+^ Tregs curtailed tumor growth without systemic autoimmunity by reducing intratumoral Treg suppressive function via an m^6^A–NF-κB axis that reduces (87). Together, these studies support the view that m^6^A readers and writers govern the balance between T-cell effector and suppressor arms in the TME, though whether to inhibit or bolster specific nodes (e.g., METTL3 *vs* YTHDF2) will likely depend on cell type and disease stage.

#### 2.2.2 Dendritic cells and cross-priming

Antigen cross-presentation by cDC1s is a bottleneck for effective CD8^+^ priming in tumors. Recent work shows that the m^6^A reader YTHDF1 is upregulated in DCs after radiotherapy via STING/IFN-I signaling and, paradoxically, restrains antitumor immunity by promoting lysosomal cathepsins, thereby degrading STING and blunting IFN-I output. DC-specific Ythdf1 loss increased type I IFN production, enhanced cross-priming, and potentiated CD8^+^ killing, improving the efficacy of radiation and combined radiotherapy plus anti-PD-L1 in multiple murine cancers; a prototype DC vaccine built from Ythdf1-deficient DCs amplified these effects (53). These findings extend earlier observations on YTHDF1-limited cross-presentation and converge on a model in which dampening YTHDF1 activity in DCs can lift constraints on antigen presentation within the tumor microenvironment (88).

#### 2.2.3 NK cells

Two independent studies place m^6^A as essential for NK-cell surveillance in cancer. Deleting METTL3 specifically in NK cells reduced their numbers and effector functions, impaired IL-15 responsiveness (via AKT–mTOR/MAPK signaling), and accelerated tumor progression in mice (89). Complementing this, loss of the reader YTHDF2 compromised NK antitumor and antiviral immunity; YTHDF2 is induced upon NK activation and supports cytotoxic programs *in vivo* (55). The cross-lab consistency here—writer and reader both required for NK fitness—underscores that, unlike some T-cell contexts, strengthening m^6^A pathways in NK cells tends to favor antitumor function.

#### 2.2.4 Myeloid cells: macrophages and MDSCs

Myeloid epitranscriptomes exert a strong influence on T-cell priming and suppression. Myeloid-specific ablation of Mettl3 increased tumor growth and metastasis and weakened the efficacy of anti-PD-1 therapy, in part by skewing macrophage polarization and altering inflammatory signaling, linking m^6^A to macrophage programming that shapes T-cell responses (90). Focusing on the eraser ALKBH5, several experimental systems indicate that m^6^A demethylation promotes immunosuppressive myeloid states: in colorectal cancer, ALKBH5 upregulated CPT1A and drove M2 polarization, facilitating tumor progression; genetic or pharmacologic interference reduced M2 programs (91). In MDSCs from colorectal cancer models, ALKBH5 downregulation elevated m^6^A and arginase-1 expression; whereas restoring ALKBH5 curtailed MDSC suppressive activity and protumor effects *in vivo* (92). Separately, radiotherapy can expand MDSCs through YTHDF2-dependent circuits, and inhibiting YTHDF2 limited MDSC accumulation and boosted radiotherapy efficacy (93). Beyond m^6^A, A-to-I editing by ADAR1 in macrophages functions as an immune brake: macrophage-specific Adar1 loss, especially when combined with IFN-γ, induced tumor regression across melanoma, lung, and colon cancer models via heightened antiviral-like signaling (PKR/eIF2α) and cytokine remodeling (94). Collectively, these studies support that tipping myeloid editing/methylation toward pro-inflammatory set points can unlock T-cell immunity, though the precise “best” lever (writer, reader, or ADAR1) may vary with therapy (radiation *vs* ICB) and tissue.

#### 2.2.5 Interplay with the TME and trafficking

Tumor environmental cues feed back on RNA-modification circuits. For example, extracellular acidosis suppressed a METTL3–m^6^A–ITGB1 axis in tumor cells to reduce CD8^+^ infiltration; restoring the axis increased T-cell entry and tumor control, illustrating how tissue pH can indirectly govern T-cell access through epitranscriptomic wiring (93). While this study probed tumor-cell METTL3, its readout—CD8^+^ infiltration and function—highlights that immune-cell behavior *in situ* reflects both immune-intrinsic and tumor-extrinsic RNA marks.

To conclude, several patterns are consistent across teams: (i) NK cells require intact m^6^A writer/reader activity for antitumor function; (ii) DC YTHDF1 restrains cross-priming, so its inhibition can be immunostimulatory; (iii) in myeloid cells, reducing ADAR1 or recalibrating m^6^A often shifts toward pro-inflammatory, T-cell-permissive states; and (iv) Treg-specific loss of YTHDF2 diminishes suppression and improves tumor control (53, 88, 93). T-cell-intrinsic METTL3 is more nuanced: some data support inhibiting METTL3 to sustain CD8^+^ function in tumors, whereas older genetic studies (outside the 5-year window) showed METTL3 supports T-cell homeostasis—suggesting disease stage, activation context, and target specificity (global *vs* cell-restricted) will determine the therapeutic direction (85).

Notably, many open questions remain to be answered. Compared with m^6^A and A-to-I editing, direct roles for m^5^C and pseudouridine in tumor-infiltrating immune cells remain scare. Early evidence ties ALKBH5/NSUN family enzymes to macrophage polarization and MDSC function, but immune-cell–specific, conditional models in solid tumors are still sparse (91). Likewise, while pseudouridine writers (e.g., PUS enzymes) and DKC1 are dysregulated in cancers, we lack definitive studies showing immune-cell-intrinsic pseudouridylation steering antitumor differentiation or function *in vivo* (95). The role of RNA modifications clearly varies with immune lineage, suggesting that context is as important as the modification itself. Rather than a universal rule, each immune subset appears to interpret RNA modifications in its own way. Current insights are largely derived from murine models, underscoring the need for clinical validation in human samples.

### 2.3 The role of RNA modifications in tumor immune evasion

#### 2.3.1 Regulation of immune checkpoint molecules (PD-1/PD-L1, CTLA-4) by RNA modifications

Across solid and hematologic cancers, converging evidence shows that epitranscriptomic programs—most prominently m^6^A and, increasingly, m^5^C—reshape checkpoint expression at the RNA level to promote immune escape. Multiple tumor models demonstrate a consistent writer–reader route in which METTL3/14 installs m^6^A on PD-L1 (CD274) transcripts and IGF2BP readers stabilize the mRNA, raising PD-L1 on the tumor surface and dampening cytotoxic T-cell activity; genetic or pharmacologic disruption of these nodes lowers PD-L1 and improves antitumor responses *in vivo*. This circuit has been shown in breast cancer (METTL3→IGF2BP3), bladder cancer (JNK–METTL3→IGF2BP1), and intrahepatic cholangiocarcinoma (where ALKBH5 demethylation maintains PD-L1), with mechanistic interventions reversing immune evasion phenotypes and, in some settings, sensitizing tumors to anti-PD-1 therapy. Together, these studies support a broadly consistent role for m^6^A as a post-transcriptional amplifier of PD-L1, executed by IGF2BP readers and modulated by demethylases in a tumor-type–dependent manner (57, 96).

Nevertheless, context clearly matters. Under hypoxia, HIF-1α can drive FTO expression, and recent work in breast cancer shows that FTO feeds a YTHDF3/PDK1–AKT–STAT3 axis to further elevate PD-L1; dual inhibition of FTO and PDK1 enhances CTL function and strengthens the effect of PD-(L)1 blockade in preclinical models. In colorectal cancer, by contrast, FTO protein can be down-regulated and associate with poor prognosis, underscoring that FTO’s checkpoint consequences are not uniform across tissues. These tumor-type- and microenvironment-dependent results explain why some datasets report strong PD-L1 suppression upon m^6^A-pathway inhibition, whereas others observe partial or pathway-specific effects, particularly under metabolic stress (97, 98).

Beyond m^6^A, m^5^C has moved from correlative to causal evidence. In non-small-cell lung cancer, the NSUN2/ALYREF axis deposits m^5^C and promotes nuclear export/stability of PD-L1 mRNA, increasing PD-L1 abundance and facilitating T-cell evasion; perturbing NSUN2 or ALYREF reduces PD-L1 and restores antitumor immunity in mouse and human systems. Thus, checkpoint control is expanded to a second modification class and aligns with broader observations that RNA-processing factors (e.g., ALYREF) act as effectors of modification-encoded export programs (81, 99).

Immune-cell–intrinsic m^6^A programs further shape checkpoint biology from the host side. In regulatory T cells (Tregs), the m^6^A reader YTHDF2 maintains fitness under inflammatory stress; its deletion reduces intratumoral Treg survival and diminishes PD-1-high expressing Treg populations, unleashing CD8^+^ responses and slowing tumor growth. In dendritic cells, YTHDF1 dampens cross-priming and type-I IFN output; loss or inhibition of YTHDF1 improves antigen presentation and enhances the efficacy of radiation plus anti-PD-L1 in multiple mouse models. Notably, tumor-intrinsic YTHDF1 depletion can elevate PD-L1 levels *in vivo* yet still sensitize tumors to immune attack—an apparent inconsistency that likely reflects dominant effects on the antigen-presentation axis and T-cell priming that outweigh PD-L1 upregulation per se (87, 88).

For PD-1 on tumor cells, direct m^6^A-site–resolved evidence remains thinner than for PD-L1. The best-supported recent theme is that demethylases modulate checkpoint programs under stress: ALKBH5 deletion in tumors remodels lactate metabolism and the suppressive myeloid/Treg milieu to boost anti-PD-1 responses, while FTO can raise PD-L1 (and, in some contexts, PD-1) through hypoxia-responsive pathways. These results are directionally consistent with the idea that lowering m^6^A demethylation (i.e., preserving m^6^A marks) tends to reduce immune suppression, but they also highlight heterogeneity across lineages and niches that complicates one-size-fits-all predictions (42).

Finally, CTLA-4 regulation by RNA modifications remains less settled. Meta-analyses and multi-omics surveys link m^6^A regulator abundance to CTLA-4 expression patterns across cancers, and Treg- and DC-centric m^6^A programs plausibly alter the outcomes of CTLA-4 blockade via effects on antigen presentation and Treg stability. However, definitive site-level maps of m^6^A (or m^5^C/Ψ) on CTLA4 mRNA, with matched reader dependencies and perturbation-rescue experiments in primary tumors, are largely absent; this is a clear gap compared with the PD-L1 literature (100).

Multiple studies now point to m^6^A-dependent stabilization of PD-L1 as a recurring route of immune escape. The strongest consensus so far is that RNA modifications can directly reinforce checkpoint pathways such as PD-L1. However, findings regarding FTO remain contradictory, as it promotes PD-L1 expression in some cancers but appears suppressed in others. Such disparity underscores how tumor type and microenvironment dictate modifier outcomes. Direct mechanistic evidence for CTLA-4 regulation remains limited, marking a significant gap in the field.

#### 2.3.2 The impact of RNA modifications on tumor antigen presentation pathways

As outlined in Section 2.1.3, dynamic RNA modifications—most prominently m^6^A—govern RNA fate through writer–reader–eraser circuits. When applied to antigen presentation, these same circuits modulate both tumor-cell immunogenicity and the priming capacity of professional antigen-presenting cells (APCs), often with node- and context-specific consequences. Tumor-intrinsic studies provide convergent, mechanistic evidence that the m^6^A reader YTHDF1 curtails antigen visibility: loss of YTHDF1 in cancer cells limits the translation of lysosomal genes, reduces lysosomal proteolysis of MHC-I and tumor antigens, upregulates surface MHC-I, and converts immunologically “cold” tumors into “hot” ones that respond to checkpoint blockade; notably, anti–PD-L1 or anti–CTLA-4 co-therapy is markedly more effective when YTHDF1 is disrupted, underscoring a causal link between m^6^A-readout and antigen presentation–driven immunity. These findings are internally consistent across multiple *in vivo* assays and single-cell analyses, although they also reveal a nuance: tumor-intrinsic YTHDF1 loss can increase PD-L1 *in vivo* while still enhancing T-cell–mediated control, implying that improved antigen presentation and T-cell priming can dominate over incremental checkpoint upregulation (88).

APC-intrinsic data reach a complementary conclusion from a different angle. In dendritic cells (DCs), YTHDF1 expression is induced by ionizing radiation through STING/type I IFN signaling and then acts in a negative feedback loop by elevating cathepsin abundance to accelerate STING degradation, dampening IFN-I release, cross-presentation of cell-associated antigens, and CD8^+^ T-cell priming. Genetic deletion or pharmacologic inhibition of YTHDF1 in DCs restores cross-priming and augments radiotherapy and radio-immunotherapy efficacy; in patients receiving stereotactic body radiotherapy, higher YTHDF1 in circulating DCs correlates with worse progression-free survival. These results replicate and extend earlier observations that YTHDF1 restrains DC cross-presentation, now tying the effect to a defined STING–lysosome axis and to human clinical samples (53).

Not all m^6^A interventions, however, move in the same direction—either experimentally or therapeutically. In endometrial cancer, the writer METTL3 enhances immunosurveillance by stabilizing the MHC-I transactivator NLRC5, thereby sustaining antigen-presentation gene expression; mechanistically, METTL3-installed m^6^A prevents YTHDF2-mediated decay of NLRC5 mRNA, and METTL3 overexpression increases intratumoral CD8^+^ T-cell infiltration and tumor control. By contrast, autophagy protein LC3 can bind NLRC5 and dampen the NLRC5/MHC-I axis independently of RNA modification, illustrating that RNA-encoded control is embedded within larger proteostasis circuits that also tune antigen presentation. Taken together, these observations indicate that the impact of m^6^A on tumor immunogenicity hinges less on overall mark abundance than on which reader or transcript lies downstream (e.g., YTHDF1–lysosome versus YTHDF2–NLRC5) (101, 102).

A second source of apparent discrepancy is cell type. Foundational work showed that METTL3 in DCs supports translation of CD40, CD80, and TLR adaptors, thereby licensing T-cell priming; multiple recent syntheses echo these findings and caution that indiscriminate writer inhibition could impair DC maturation and co-stimulation even as YTHDF1 blockade enhances cross-presentation. In short, the specific node targeted matters: targeting a pro-presentation brake (YTHDF1) in DCs or tumors may be beneficial, whereas broad reduction of m^6^A in DCs can blunt antigen presentation despite potential gains elsewhere (54, 103).

Beyond m^6^A, direct, site-resolved evidence that m^5^C, m^1^A, or Ψ regulate the MHC-I/MHC-II machinery or cross-presentation remains limited; recent reviews highlight these marks as plausible modulators of APC programs and tumor immunogenicity, but definitive experiments—mapping modification sites on B2M, HLA/H2 heavy chains, TAP1/2, TAPBP, ERAP/ERAAP, and cross-presentation genes, with matched reader dependencies and perturbation–rescue in primary tumors or human DCs—are still rare. By contrast, the m^6^A space now includes multiple orthogonal demonstrations (genetics, proteomics, single-cell profiling, functional vaccination) that connect reader activity to antigen processing, MHC-I stability, and response to immunotherapy (47, 53, 88).

Open questions emerge from these comparisons. First, consistency is high for YTHDF1 as a brake on antigen visibility in both tumors and DCs, yet the magnitude and dominant downstream pathway (lysosomal proteolysis *vs*. STING erosion) differ by compartment and stimulus (e.g., radiation), implying that context-specific targeting will be essential. Second, writer effects diverge: METTL3 can promote antigen presentation via NLRC5 in tumor cells but is also required for DC co-stimulation—raising the practical challenge of cell-type–selective delivery or reader-focused strategies. Third, base-resolved modification maps on canonical antigen-presentation transcripts in human tumors and APCs are incomplete, and how reader competition (IGF2BPs *vs*. YTHDFs) is wired on these RNAs *in vivo* is not fully defined. Finally, most positive *in vivo* data derive from genetic perturbation; drug-like inhibitors with proven on-target engagement for readers/writers in tumors or DCs, and their combinability with radiotherapy or vaccines, remain in early stages. Addressing these gaps with time-resolved, cell-state–resolved epitranscriptomic profiling under defined cues (IFN-γ, hypoxia, radiation) and rigorous perturbation–rescue will be key to translating mechanism into durable patient benefit (53, 88, 101).

#### 2.3.3 RNA modification–mediated tumor cell stress adaptation and immune resistance

Building on Section 2.3.2, stressors typical of the tumor microenvironment—hypoxia, acidosis, nutrient scarcity, and oxidative damage—reconfigure epitranscriptomic circuits and, in turn, reshape how tumors withstand immunity. A consistent theme across models is that hypoxia-driven programs remodel m^6^A dynamics: HIF-1α can transcriptionally induce the demethylase FTO, which elevates PD-L1 via a PDK1–AKT–STAT3 cascade and blunts T-cell attack; pharmacologic or genetic reduction of FTO in hypoxia restores sensitivity to PD-L1 blockade in preclinical breast cancer, directly linking a stress sensor to immune resistance. Earlier genetic work on the related demethylase ALKBH5 complements this picture from a metabolic angle: ALKBH5 loss lowers lactate accumulation, curtails suppressive myeloid/Treg niches, and markedly augments anti-PD-1 efficacy *in vivo*. Together, these studies agree that hypoxic conditions favor demethylase-driven immune evasion, while demethylase inhibition counters it; differences largely reflect tissue context and which downstream readers and pathways (e.g., STAT3 versus metabolic rewiring) are engaged (42, 98).

Stress adaptation also intersects with antigen visibility through m^6^A readers that tune proteostasis. In tumor cells, YTHDF1 enhances translation of the lysosomal/acidic hydrolase genes, promoting antigen and MHC-I turnover; deleting YTHDF1 stabilizes MHC-I at the surface, improves CD8^+^ priming, and heightens responsiveness to checkpoint blockade in multiple mouse systems. Intriguingly, this occurs even when PD-L1 rises modestly *in vivo*, implying that improved antigen presentation can dominate over incremental checkpoint upregulation in determining treatment outcome. Under therapy-induced stress, dendritic cells mount a related but mechanistically distinct response: ionizing radiation drives a STING/type-I IFN burst that is subsequently dampened by YTHDF1-dependent cathepsin upregulation and STING degradation; ablating YTHDF1 in DCs preserves IFN-I, rescues cross-presentation, and strengthens radio-immunotherapy, with patient data showing higher circulating DC-YTHDF1 correlates with poorer control. These tumor-intrinsic and APC-intrinsic datasets are directionally consistent—YTHDF1 acts as a brake on anti-tumor immunity—while revealing pathway differences (lysosome–MHC turnover versus STING erosion) that depend on cell type and stimulus (53, 88).

Oxidative stress and lipid peroxidation introduce a second layer, where m^6^A programs steer susceptibility to ferroptosis, a death pathway now linked to therapy response. Multiple cancer studies show that METTL3-installed m^6^A marks on SLC7A11 can be read by IGF2BP proteins to stabilize the cystine transporter transcript, elevate glutathione synthesis, and suppress ferroptosis, and FTO likewise protects colorectal tumors by sustaining SLC7A11/GPX4 expression. Yet this axis is not unidirectional: contexts exist in which ALKBH5 erases m^6^A on ferroptosis regulators (e.g., SLC7A11, GPX4) and thereby promotes lipid-peroxidation–driven death, underscoring lineage- and stress-state–specific wiring of writer/eraser–reader pairs. Reviews synthesizing these primary datasets further connect ferroptosis control to immune and radiotherapy responsiveness, although definitive in-patient evidence that m^6^A-ferroptosis rewiring alone improves immunotherapy outcomes remains limited (44, 103–106).

Beyond these pathways, stress granules (SGs) provide an adaptable harbor for mRNAs during acute insults; recent work indicates that YTHDF2 modulates SG stability through G3BP1 in an m^6^A-dependent manner, influencing how quickly translation resumes when stress abates. While these data clarify a mechanistic role for readers in stress partitioning, their direct contribution to immune resistance is still circumstantial, and prior single-molecule studies have cautioned that m^6^A is not a universal gatekeeper for SG entry—pointing to a nuanced, transcript- and context-dependent model that needs disease-relevant validation. Hypoxia-coupled m^6^A reprogramming of metabolic effectors also extends to glycolysis (e.g., YTHDF2-stabilized HDAC4 sustaining glycolytic flux in pancreatic cancer under low oxygen), linking survival metabolism to dampened immunogenicity through both antigen presentation and checkpoint axes (107).

Several knowledge gaps remain. First, stress-conditioned, base-resolved maps for canonical immune-control transcripts (e.g., CD274/PD-L1, B2M, TAP1/2, GPX4, SLC7A11) in patient tumors and intratumoral APCs are sparse, limiting causal assignment of reader competition under hypoxic or nutrient stress. Second, demethylase effects on immunity are broadly consistent under hypoxia (FTO/ALKBH5 favoring resistance), yet diverge across lineages and stressors in ferroptosis control, suggesting that the same enzyme can be pro- or anti-immunogenic depending on which targets are methylated and which readers predominate. Third, most positive studies rely on genetics; drug-like, reader-selective inhibitors with verified on-target activity *in vivo* are early-stage, and their combinability with radiotherapy, ferroptosis inducers, or checkpoint blockade, needs systematic testing. Finally, stress-translation coupling remains debated—some earlier models posited widespread 5′-UTR m^6^A-driven cap-independent initiation during the integrated stress response, whereas more recent ribosome-centric work argues for context-limited effects—so careful reconciliation with disease settings is warranted (98, 108).

### 2.4 Interaction between RNA modifications and tumor microenvironment

#### 2.4.1 The impact of RNA modifications on immune cell infiltration in the tumor microenvironment

Building on Sections 2.3.2–2.3.3, a convergent body of *in vivo* work now shows that RNA modifications—most clearly m^6^A decoded by YTH-family and IGF2BP readers—reconfigure which immune populations can access, or are excluded from tumors. Tumor-intrinsic loss of the m^6^A reader YTHDF1 converts “immune-desert” lesions into T cell–inflamed tumors by curbing lysosomal translation, stabilizing MHC-I on the surface, and enhancing antigen persistence; these changes coincide with broader remodeling of the tumor microenvironment (TME) with increased CD8^+^ T-cell infiltration and improved checkpoint response in mouse models. Human single-cell datasets in the same study align with this pattern. In professional antigen-presenting cells, YTHDF1 performs a parallel—yet mechanistically distinct—function: ionizing radiation induces a STING/type-I IFN burst that is then dampened by YTHDF1-driven cathepsin upregulation and STING degradation; deleting or inhibiting YTHDF1 in dendritic cells preserves cross-priming capacity and augments CD8^+^ infiltration after radiotherapy or radio-immunotherapy, with patient correlative data reinforcing in the same direction. The overall message is consistent across compartments—YTHDF1 acts as a brake on T-cell entry and activation—even though the dominant downstream pathway (lysosomal antigen/MHC-I turnover in tumor cells versus STING erosion in DCs) differs by cell type and stimulus (53, 88).

Demethylases link metabolic stress to immune exclusion and myeloid recruitment. ALKBH5 tunes the lactate efflux program (via MCT4/SLC16A3), increasing local acidosis and favoring the accumulation of Tregs and MDSCs; genetic deletion or small-molecule inhibition of ALKBH5 reduces these suppressive infiltrates and improves anti-PD-1 efficacy in multiple syngeneic models, directly tying an m^6^A eraser to the composition of tumor-infiltrating immune cells. These results are internally replicated across orthogonal rescue experiments (including MCT4 re-expression) and are among the clearest causal examples that an epitranscriptomic enzyme can “dial” the TME toward or away from immunosuppression (42).

Chemokine circuits supply a second, tumor-intrinsic route from m^6^A to infiltration phenotypes, but here the direction of effect is context-dependent. In colorectal cancer, targeting METTL3 reprograms the TME by lowering pro-tumor chemokines (CXCL1, CXCL5, CCL20) and enhancing antitumor responses, consistent with a METTL3→chemokine axis that favors myeloid recruitment and immune evasion. By contrast, in endometrial cancer, METTL3 safeguards NLRC5 via a YTHDF2-dependent mechanism, maintaining MHC-I transcriptional programs and associating with greater CD8^+^ T-cell presence and tumor control. Read together, these studies support a coherent principle—which targets are marked and which reader circuit is engaged determine whether m^6^A promotes or restrains immune infiltration—and they caution against one-size-fits-all assumptions across tissues (101, 109).

Innate compartments also participate. In tumor-associated macrophages (TAMs), YTHDF2 promotes a protumoral polarization state and suppresses antigen-presentation programs; myeloid-specific Ythdf2 loss reprograms TAMs toward antitumor phenotypes and enhances CD8^+^-mediated control *in vivo*. Complementing this, tumor-intrinsic YTHDF2 has been shown to limit expression of the chemokine CX3CL1, thereby restraining macrophage recruitment and shaping the myeloid landscape within tumors. These macrophage-focused datasets are highly consistent in demonstrating that YTHDF2 tilts the innate niche toward immune suppression, although the proximal targets (STAT1/interferon signaling *vs*. CX3CL1) vary by model and compartment (110, 111).

Despite this progress, several tensions and gaps remain. First, directionality diverges for METTL3 across cancer types: in CRC models it amplifies chemokines linked to myeloid influx, while in endometrial tumors it preserves NLRC5-driven MHC-I and correlates with higher CD8^+^ content—differences likely rooted in target selection and reader competition that are not yet mapped at base-resolution in primary human specimens. Second, most demethylase data are strongest for ALKBH5 in the setting of lactate-rich TMEs; whether analogous infiltration effects extend broadly to FTO or to non-hypoxic contexts requires direct testing. Third, although multiple studies quantify changes in CD8^+^, Treg, MDSC, and TAM populations after manipulating m^6^A nodes, causal chemokine targets (e.g., CXCL9/10, CXCL1, CX3CL1) have not been uniformly validated with site-resolved epitranscriptomic maps and reader-swap rescue. Finally, translational chemistry is early: reader-selective inhibitors with verified on-target engagement in tumors or dendritic cells—and prospective trials powered on infiltration endpoints—remain to be developed. Addressing these issues will require cell-type–resolved, time-aware mapping of modifications on chemokine and checkpoint transcripts under defined cues (IFN-γ, hypoxia, radiation), paired with perturb-and-rescue designs and human tissue validation (42, 101, 109).

#### 2.4.2 Interaction between RNA modifications and cancer-associated fibroblasts

Converging evidence now anchors RNA modifications—chiefly m^6^A—as active currency in CAF–tumor communication rather than background noise. Two mechanistic routes recur across models. First, CAFs can raise m^6^A activity inside tumor cells without necessarily transferring RNA: in non-small cell lung cancer (NSCLC), CAF-conditioned cues (notably VEGFA) induce tumor-intrinsic METTL3, elevating m^6^A on RAC3 and driving AKT/NF-κB signaling, invasion, and *in vivo* growth. This establishes a paracrine CAF→tumor METTL3→m^6^A–RAC3 axis with genetic and animal-level support. Second, CAFs can export the writer itself: multiple studies show METTL3 packaged in CAF exosomes enters cancer cells to install m^6^A on metabolic transcripts—SLC7A5 (LAT1) in NSCLC and ACSL3 in colorectal cancer (CRC)—thereby stabilizing targets, reprogramming glutamine/FA metabolism, suppressing ferroptosis, and accelerating metastasis; knockdown of exosomal METTL3 curtails tumor growth *in vivo*. Together, these data are internally consistent in positioning METTL3 as a stromal lever of cancer metabolism and progression, while also revealing mechanistic diversity in the immediate tumor-cell targets (RAC3 *vs*. SLC7A5 *vs*. ACSL3) and in the mode of delivery (paracrine induction *vs*. exosomal transfer) (112, 113).

These stromal circuits extend beyond m^6^A and beyond direct tumor proliferation. In pancreatic cancer, CAF-derived extracellular vesicles carry PIAT, a factor that drives m^5^C modification in recipient cells and promotes neural remodeling—one of several demonstrations that stromal vesicles can deliver functional RNA-modifying capacity across cell types. Meanwhile, independent clinical-pathologic studies show PD-L1 can be expressed by CAFs and that CAFs upregulate PD-L1 in tumor cells via AKT phosphorylation, reinforcing the idea that stromal programs help enforce immune suppression. Notably, while m^6^A clearly governs PD-L1 expression in multiple tumor-intrinsic contexts, direct causal links from CAF m^6^A machinery to tumor PD-L1 are still sparse; where PD-L1 rises after CAF–tumor crosstalk, the current best-supported mechanisms are AKT signaling and miRNA-bearing exosomes, with m^6^A often acting upstream on metabolism and stress adaptation that secondarily shapes immunosuppressive tone. This distinction matters for therapy design (111, 114).

Angiogenesis and matrix remodeling add further layers to how CAF epitranscriptomics sculpt the tumor microenvironment. FTO in CAFs has been shown to erase m^6^A on multiple pro-angiogenic transcripts, preventing the YTHDF2-mediated decay and promoting neovascularization; pharmacologic or genetic inhibition reverses these effects, arguing that stromal erasers can be as consequential as writers. More broadly, the CAF compartment is heterogeneous, with myofibroblastic (myCAF), inflammatory (iCAF), and additional spatially conserved states captured by single-cell and spatial atlases across tumor types. Yet we still lack state-resolved, base-level maps of RNA modifications within CAF subtypes, making it unclear whether iCAFs preferentially export writers while myCAFs rely on paracrine induction of tumor METTL3—or whether specific readers (IGF2BPs *vs*. YTHDFs) dominate in each niche. Current single-cell atlases set the stage but do not resolve which modified transcripts underpin CAF-specific functions in human tumors (113, 115).

Across studies, consistencies are striking: (i) CAF interventions that increase functional m^6^A in cancer cells tend to enhance invasiveness, metabolic plasticity, ferroptosis resistance, and, indirectly, immune evasion; (ii) exosomal delivery of METTL3 is sufficient to rewire tumor metabolism and growth; and (iii) targeted disruption of these stromal–epitranscriptomic nodes restrains tumor progression *in vivo*. Differences arise in the immediate outputs—glutaminolysis via SLC7A5 in NSCLC, fatty-acid activation via ACSL3 in CRC, and pro-migratory signaling via RAC3—and in whether CAFs act primarily by secreting a writer versus inducing tumor writers through growth-factor signaling. For immune checkpoints, CAFs clearly raise PD-L1 through AKT and can themselves express PD-L1, but the m^6^A-specific CAF→PD-L1 link remains less complete than the metabolism and ferroptosis stories. Addressing these discrepancies will require paired, cell-type-resolved modification maps (CAF and tumor) under defined cues, combined with reader-swap and target-site mutagenesis to test causal wiring in patient-derived models (116, 117).

#### 2.4.3 Regulation of angiogenesis and immunosuppressive cytokines by RNA modifications

Building on the preceding discussion of immune infiltration and stromal crosstalk, a converging theme is that epitranscriptomic control—most prominently m^6^A—recalibrates both the vascular program and the cytokine milieu in tumors. Mechanistically, well-substantiated work in lung cancer shows that m^6^A deposited within the 5′UTR of VEGFA enables cap-independent translation through a YTHDC2/eIF4GI–dependent mechanism, boosting VEGF-A production and angiogenesis *in vitro* and *in vivo*; genetic and biochemical perturbations in this axis curb neovascularization and tumor growth, establishing causality rather than correlation. Complementing this, m^6^A “reader” IGF2BP proteins stabilize pro-angiogenic transcripts such as VEGFA and EPHA2, and exosomal transfer of IGF2BP2 from tumor cells can activate endothelial PI3K–AKT signaling to drive vessel formation and metastatic spread. Together, these studies argue that m^6^A commonly tilts the balance toward pro-angiogenic output at multiple regulatory tiers (translation, stability, intercellular transfer) (118–120).

These vascular effects interface tightly with immunoregulation. VEGF-A itself is increasingly recognized as an immunomodulator that dampens antitumor lymphocyte function and antigen presentation, providing a direct link between neovascular cues and immune suppression. In parallel, tumor-conditioned myeloid cells undergo epitranscriptomic reprogramming: lactate accumulation in the TME induces METTL3 via protein lactylation, which enhances m^6^A-JAK1–STAT3 signaling in tumor-infiltrating myeloid cells and reinforces immunosuppressive transcriptional programs. Separately, IL-10–STAT3 signaling upregulates the m^6^A reader YTHDF2 in tumor-associated macrophages, and restraining YTHDF2 reprograms macrophages toward an IFN-high, antigen-supportive state and restores CD8^+^ T-cell activity. Although these reports interrogate different arms of the pathway (writer *vs* reader), they consistently position m^6^A machinery as a rheostat for the cytokine environment and myeloid function in tumors (110, 121, 122).

Across indications, experimental concordance is strongest for a pro-angiogenic role of the m^6^A system: upregulating VEGF-A translation (lung cancer) and stabilizing additional pro-vascular or pro-metastatic transcripts (e.g., EPHA2; HDGF), with endothelial activation amplified by m^6^A-programmed RNA cargo in tumor exosomes. Yet there are notable context-dependent exceptions. In colorectal cancer, m^6^A -dependent circuits can also limit vascularization via specific reader–lncRNA interactions (e.g., a YTHDF1–LINC01106 axis reported to suppress vascular generation), and in hepatocellular carcinoma or renal cancer, YTHDF2 has been linked to vessel normalization by promoting decay of angiogenic factors—findings that remind us readers can execute opposing outcomes depending on target sets and cellular compartment. On the immunologic side, lactate-driven METTL3 in myeloid cells fosters suppression, whereas myeloid METTL3 has also been reported to support anti-tumor responses under different cues, echoing a broader pattern in which cell type, metabolic state, and inflammatory tone determine whether m^6^A skews toward immune evasion or activation. These divergences likely reflect differences in tumor lineage, hypoxia and nutrient stress, and the reader repertoire available in a given niche (119, 120, 123).

Beyond m^6^A, evidence for other marks is emerging but less uniform. m^5^C pathways (NSUN2–ALYREF) can stabilize growth factor and EGFR–STAT3 transcripts and have been linked to pro-tumor signaling and immune evasion in hepatocellular carcinoma; whether these changes directly rewire tumor angiogenesis or cytokine secretion *in vivo* at scale is still being clarified. Current data therefore support a model in which multiple RNA marks intersect with hypoxia/HIF and STAT3 circuits to tune both vascular cues and immunosuppressive cytokines, but the weight of causal evidence remains stronger for m^6^A (124).

Several knowledge gaps merit emphasis. First, we still lack single-cell, *in situ* maps of mark-specific sites on cytokine mRNAs (e.g., IL10, TGFB1) across human tumors under therapy to unambiguously assign direct versus secondary effects. Second, the field needs systematic testing whether targeting specific m^6^A nodes (e.g., YTHDF2 in TAMs or IGF2BP2/3 at the tumor–endothelium interface) leads to vessel normalization and improved antigen trafficking—alone or combined with anti-VEGF and checkpoint blockade. Third, how exosomal m^6^A regulators or modified RNAs traffic among CAFs, endothelial cells, and myeloid subsets to create localized cytokine/angiogenic niches remains incompletely defined. Carefully controlled, longitudinal studies that integrate epitranscriptomic profiling with metabolite, cytokine, and vascular phenotyping will be critical to translate these mechanistic insights into durable immuno-vascular therapies (120, 121).

## 3 The role of RNA modifications in tumor treatment resistance

### 3.1 Mechanisms of chemoresistance mediated by RNA modifications

RNA modifications reshape drug response by rewiring RNA fate at multiple control points—mRNA stability, translation, RNA processing, and RNA–protein interactions—thereby tuning ferroptosis sensitivity, DNA damage repair, drug efflux, tumor stemness, and niche dependence. Among these, m^6^A and ac^4^C currently have the strongest experimental support in chemoresistance, with mounting evidence for m^5^C and A-to-I editing.

A recurring theme is that m^6^A edits on ferroptosis gatekeepers (e.g., SLC7A11) blunt lipid peroxidation and protect tumor cells from chemotherapy-induced death. In patient-derived bladder cancer models, early cisplatin resistance emerges with reduced m^6^A on SLC7A11, diminished YTHDF3 binding, slower mRNA decay, and elevated SLC7A11 protein, collectively suppressing ferroptosis and enhancing survival; these dynamics are seen within 48h of cisplatin exposure in lines and organoids (112, 125–127). Mechanistically related observations extend to other systems where m^6^A writers/readers stabilize SLC7A11 to maintain antioxidant capacity, although the exact reader (YTH family *vs*. IGF2BPs) and direction of effect can be context-dependent (128).

m^6^A also hardwires DDR, thereby modulating sensitivity to DNA-damaging chemotherapy. In breast cancer, METTL3 promotes homologous recombination (HR) via the EGF–RAD51 axis; METTL3 loss impairs HR and sensitizes cells to doxorubicin, while YTHDC1 reads the modification to protect HR-related transcripts (125). Complementary data show YTHDF1 and METTL14 coordinate S-phase entry and HR factor expression; knocking them down increases γH2AX foci and sensitizes cells to adriamycin/cisplatin/Olaparib (126). In gastric cancer, METTL3 knockdown suppresses DNA repair pathways and augments oxaliplatin sensitivity, again linking m^6^A to chemoresponse through DDR attenuation (124). These studies converge on a model in which m^6^A writers/readers bolster HR and checkpoint signaling to withstand genotoxic chemotherapy; however, which specific m^6^A-programmed DDR transcripts dominate is tumor-type specific and remains to be comprehensively mapped.

At the cell membrane, m^6^A can increase efflux pump expression. In colorectal cancer, IGF2BP3 binds m^6^A-modified ABCB1 (MDR1) mRNA, stabilizes it, and triggers multidrug resistance; genetic perturbation of IGF2BP3 decreases ABCB1 and restores chemosensitivity (129). In breast cancer, METTL3 cooperates with IGF2BP3 to stabilize HYOU1, increasing doxorubicin resistance *in vitro*, silencing either component reverses resistance (130). These efflux/metabolic axes are consistent with—but more mechanism-resolved than—earlier correlative reports connecting m^6^A readers to chemoresistance (131).

m^6^A demethylases can sustain stemness traits that underlie refractory disease. In triple-negative breast cancer (TNBC), ALKBH5 demethylates FOXO1 mRNA to support cancer stem-cell properties and doxorubicin resistance; ALKBH5 depletion reduces stemness and resensitizes cells (132). In parallel, KIAA1429/VIRMA enhances FOXM1 mRNA stability via YTHDF1 to drive cisplatin resistance in gastric cancer, and its knockdown re-sensitizes resistant xenografts (133). Together, these studies point to convergent m^6^A wiring of stemness transcriptional programs (FOXO1/FOXM1), though which demethylase *vs*. writer predominates varies by lineage.

Chemoresistant clones often exploit protective niches. In AML, METTL3 increases m^6^A on ITGA4, extending ITGA4 mRNA half-life and elevating integrin α4 protein to enhance bone-marrow homing/engraftment and drug tolerance. Importantly, the METTL3 inhibitor STM2457 reverses homing and chemoresistance *in vivo*, translating epitranscriptomic modulation into a therapeutic gain (127). These data provide a causative link between m^6^A, microenvironmental retention, and clinical resistance—a connection likely relevant beyond AML but still underexplored in solid tumors.

N^4^-acetylcytidine (ac^4^C), catalyzed by NAT10, stabilizes subsets of mRNAs to promote drug resistance. In melanoma, NAT10 is upregulated in dacarbazine-resistant cells/patient samples and installs ac^4^C on DDX41 and ZNF746 transcripts; genetic or pharmacologic NAT10 inhibition (Remodelin) resensitizes cells and reduces tumor burden in mouse models (134). For m^5^C, NSUN2 upregulation confers ferroptosis resistance in esophageal cancer, linking m^5^C-dependent RNA stability to therapy tolerance—an emerging theme likely to extend to chemoresistance in other gastrointestinal tumors (135).

A-to-I editing by ADAR1 can reprogram metabolism and stress responses under chemotherapy. In gastric cancer patient-derived organoids, ADAR1 editing of the SCD1 3′UTR increases KHDRBS1 binding and mRNA stability, boosting lipid droplet formation to buffer ER stress and drive 5-FU + cisplatin resistance; SCD1 inhibition reverses these effects *in vivo* (136). These data underscore that non-methyl modification systems also sculpt chemoresponse.

Across tumor types, independent groups consistently report that: (i) m^6^A writers/readers stabilize pro-survival transcripts (SLC7A11, HR factors, FOXM1/FOXO1, ABC transporters) to promote resistance; (ii) demethylases (e.g., ALKBH5) reinforce stemness and drug tolerance; (iii) niche-dependence can be m^6^A-programmed (ITGA4). Yet results are context-dependent. For example, the same regulator can either sensitize or desensitize depending on dominant targets, reader usage (YTH *vs*. IGF2BP families), and therapy class. Reports mapping m^6^A to ferroptosis sometimes implicate different readers (YTHDF3 *vs*. YTHDF1/IGF2BP2/3), and not all studies agree on whether increased m^6^A on SLC7A11 promotes or suppresses ferroptosis—likely reflecting cell-type-specific positioning of m^6^A peaks and reader availability. Moreover, while DDR reinforcement by METTL3/YTHDC1 is robust in breast cancer and gastric cancer models, the exact HR targets and their clinical predominance remain to be defined across tumor lineages. Finally, non-m^6^A marks (ac^4^C, m^5^C) clearly influence resistance in select settings, but their transcriptome-wide target repertoires in human tumors remain sparsely charted.

### 3.2 The relationship between RNA modifications and radiotherapy resistance

Radiotherapy (RT) efficacy is shaped by two interlocking axes: intrinsic DNA damage response (DDR) programs within irradiated tumor cells and extrinsic RT-elicited innate and adaptive immunity in the tumor microenvironment (TME). Across both axes, epitranscriptomic regulation has emerged as a determinant of radioresponse, with recent experimental studies showing that dynamic RNA modifications (principally m^6^A, m*^5^*C, and A-to-I editing) rewire DNA repair capacity, ferroptosis sensitivity, and STING/type I interferon (IFN-I) signaling. Together, these layers can either entrench radioresistance or create opportunities for radiosensitization.

On the tumor-intrinsic side, m^6^A demethylation by ALKBH5 has now been causally tied to DDR proficiency and RT resistance in glioblastoma stem-like cells (GSCs). A 2025 Theranostics study identified a radiation-responsive MST4–USP14–ALKBH5 axis that stabilizes ALKBH5 protein, enhances homologous recombination (HR) repair of double-strand breaks, and confers radioresistance; pharmacologic inhibition of the deubiquitinase USP14 (IU1) disrupted ALKBH5 stabilization and improved control of GSC-derived xenografts by RT. Mechanistically, integrated RNA-seq/m^6^A-seq implicated ALKBH5-dependent regulation of HR effectors, and the authors observed pathway induction following ionizing radiation, pointing to a feed-forward “DDR-m^6^A” circuit under RT stress (137). Complementing this, work in head-and-neck squamous cell carcinoma (HNSCC) demonstrated that the m^6^A demethylase FTO is a druggable radiosensitizer: genetic or pharmacologic FTO inhibition elevated persistent γ-H2AX foci, impaired HR (reduced RAD51 foci), and enhanced tumor control by RT in human xenografts and immune-competent murine models—without worsening mucositis—thereby improving the therapeutic index of RT (138). These convergent results support a consistent picture: m^6^A erasers that preserve genome integrity after irradiation (ALKBH5, FTO) tend to promote radioresistance, and their inhibition radiosensitizes at least some tumors.

Tumor cells also use m^6^A to couple RT resistance to ferroptosis avoidance. In nasopharyngeal carcinoma (NPC), m^6^A writer METTL3 stabilized SLC7A11 mRNA, bolstered anti-ferroptotic defenses, and increased clonogenic survival under irradiation; METTL3 depletion or ferroptosis induction restored radiosensitivity *in vitro* and *in vivo*. A second NPC study found that the lncRNA HOTAIRM1 augments radioresistance by maintaining FTO acetylation and driving m^6^A-dependent alternative splicing of CD44 toward CD44v isoforms, which suppress RT-induced ferroptosis; silencing this HOTAIRM1–FTO–YTHDC1–CD44 axis resensitized tumors (139). In esophageal squamous cell carcinoma (ESCC), m^6^A-modified lncRNA LNCAROD is stabilized and, in turn, sustains PARP1-mediated DNA repair, yielding radioresistance; genetic disruption of this lncRNA pathway radiosensitized ESCC in xenografts. Taken together, independent groups working in different epithelial cancers converge on a common theme: m^6^A programs stabilize pro-repair transcripts and/or disable ferroptotic cell death, thereby dampening RT lethality.

Beyond m^6^A, m*^5^C* methylation also shapes radioresponse. In cervical cancer, NSUN6-mediated m*^5^*C modification of NDRG1 mRNA increases binding by the m*^5^*C reader ALYREF, stabilizes NDRG1, and enhances HR repair; *in vitro*, patient-derived organoids, xenografts, and clinical cohorts all linked high NSUN6 and elevated m*^5^*C burden with RT resistance and poorer outcomes. Genetic silencing of the NSUN6/ALYREF–m*^5^*C–NDRG1 axis increased DNA damage and restored radiosensitivity (140). These data extend a growing consensus that multiple RNA modifications converge on the common biochemical bottleneck—efficient DSB repair—to set the RT response threshold.

A-to-I RNA editing contributes a distinct, editing-dependent route to radioresistance. In NSCLC, ADAR1 promoted radioresistance by binding the E3 ligase RAD18 to facilitate PCNA monoubiquitination and DNA damage tolerance; ADAR1 depletion or pharmacologic inhibition decreased DSB repair capacity, increased γ-H2AX persistence, and sensitized tumors to RT in xenografts. These results mechanistically decouple ADAR1’s well-known immunologic effects from a direct, tumor-intrinsic DDR function relevant under irradiation.

Crucially, epitranscriptomic control of radioresponse also extends to the immune compartment. A 2024 JCI study showed that RT induces the m^6^A reader YTHDF1 specifically in dendritic cells (DCs), where YTHDF1 boosts cathepsin translation, accelerates lysosomal degradation of activated STING complexes, and blunts IFN-I production. DC-specific Ythdf1 deletion enhanced cross-priming, amplified RT-elicited CD8^+^ T cell responses, and improved tumor control by RT or radio-immunotherapy in multiple murine models; higher DC YTHDF1 in patients receiving RT associated with inferior outcomes (53). In parallel, a 2023 *Cancer Cell* study identified myeloid YTHDF2 as an RT checkpoint: irradiation upregulated YTHDF2 in tumor-infiltrating myeloid cells, preserving an immunosuppressive MDSC program and curtailing antigen presentation; conditional myeloid Ythdf2 loss reprogrammed myelopoiesis, increased DCs and macrophages with pro-inflammatory phenotypes, and synergized with RT (and PD-L1 blockade) to overcome tumor radioresistance (141). These independent lines of evidence are strikingly consistent in placing m^6^A readers as negative regulators of the STING–IFN axis and of productive antitumor immunity after RT.

In sum, tumor-intrinsic RNA-modification circuits (ALKBH5/FTO/m^6^A; NSUN6/*m*^5^C; ADAR1 editing) and immune-compartment readers (YTHDF1/YTHDF2) form a cohesive epitranscriptomic framework for radioresistance. These insights align across multiple models and disease sites, and they directly suggest translational strategies—DDR-tilting with FTO/ALKBH5 blockade, ferroptosis-permissive m^6^A programs in NPC, and reader inhibition to sustain STING–IFN signaling after RT—that merit prospective, biomarker-guided testing in combination with contemporary RT and immunotherapy.

## 4 Therapeutic strategies targeting RNA modification proteins

### 4.1 Catalytic inhibition of writers/erasers and editors

The most mature drugging efforts focus on catalytic pockets of m^6^A writers/erasers and on NAT10 (ac^4^C) or ADAR1 (A-to-I). First-in-class METTL3 inhibitors have moved beyond proof-of-concept into patients. The tool compound STM2457 validated both on-target activity and anti-leukemic efficacy in AML models, and more recently enhanced *in vivo* responses to venetoclax in resistant disease, supporting combination strategies (142–144). The oral clinical candidate STC-15 showed acceptable safety, pharmacodynamic target engagement, and early signs of activity in a phase 1 trial across solid tumors, with planned checkpoint-inhibitor combinations—an encouraging signal for translational feasibility (145).

On the “eraser” side, FTO has emerged as a radiosensitization target: genetic and pharmacologic FTO blockade increased DNA damage (reduced RAD51 foci, impaired HR) and improved tumor control by radiotherapy in HNSCC models—without exacerbating mucositis—indicating a widened therapeutic window (146). For ALKBH5, multiple groups link demethylation to treatment tolerance; while selective clinical inhibitors remain at an early stage, recent chemistry and disease biology reinforce its druggability trajectory (147, 148).

Beyond m^6^A, NAT10-mediated ac^4^C stabilizes resistance programs. In melanoma, NAT10 installs ac^4^C on *DDX41* and *ZNF746* transcripts to drive dacarbazine resistance; NAT10 inhibition (Remodelin) resensitized tumors in mice, nominating ac^4^C as a tractable axis. Related work in TNBC connected NAT10–ac^4^C to glycolysis and an immunosuppressive TME, underscoring immuno-oncology combination potential (134). ADAR1 (A-to-I) is also advancing: multiple preclinical efforts now report small-molecule ADAR1 inhibitors that boost MDA5-dependent IFN signaling and show antitumor activity, while orthogonal data in prostate cancer describe ZYS-1 with *in vivo* efficacy—collectively arguing that pharmacologic ADAR1 blockade is becoming feasible, though clinical translation is still ahead (149).

### 4.2 Targeted protein degradation of RNA-modification enzymes

Because writers/erasers act within multi-protein machines, degradation can outperform active-site inhibition. Multiple 2024 studies report PROTACs that remove METTL3–METTL14 more effectively than parent inhibitors, with more profound m^6^A loss and stronger anti-leukemic effects *in vitro* and *in vivo*. Parallel efforts identified VHL- and CRBN-recruiting degraders (e.g., WD6305, KH-series) that broaden the chemotype space (143, 150). Analogously, FTO degraders have been disclosed, including a proof-of-concept PROTAC and a ligand-induced degradation mechanism using vitamin E succinate via the DTX2/UFD1 pathway—together suggesting multiple routes to extinguish FTO function beyond occupancy.

Independent discovery tracks converge on the idea that degrading m^6^A enzymes can produce larger phenotypic effects than partial enzymatic inhibition. Outstanding issues include degrader selectivity across METTL family members, in-tumor exposure, and whether deeper m^6^A suppression compromises antitumor immunity in combination regimens.

### 4.3 Reader antagonism and immune re-programming

Targeting readers can reprogram the immunologic landscape without directly altering global modification stoichiometry. In dendritic cells, RT induces YTHDF1, which enhances cathepsin translation and accelerates lysosomal degradation of activated STING complexes; DC-specific Ythdf1 loss boosts IFN-I, cross-priming, and tumor control after RT or radio-immunotherapy. Myeloid YTHDF2 likewise limits RT efficacy by preserving MDSC programs; Ythdf2 loss reprograms myelopoiesis, increases inflammatory myeloid subsets, and synergizes with RT/anti-PD-L1. These two studies—conducted in different teams, models, and lineages—are concordant in positioning m^6^A readers as immunologic brakes in the RT context (53).

For oncogenic readers, IGF2BP family inhibitors are emerging. BTYNB disrupted IGF2BP1–mRNA binding and induced leukemic differentiation; more recently, IGF2BP1 blockade reduced YAP1 signaling and tumor growth, strengthening the case for the druggability of reader–RNA interfaces (151). As a complementary modality, siRNA delivery against YTHDF1 using dual-targeted, photothermal chromium nanoparticles reprogrammed TAMs toward M1, increased CD8^+^ infiltration, and suppressed liver tumors *in vivo*, illustrating targeted reader knockdown as an immunotherapy.

### 4.4 Precision epitranscriptome re-wiring (RNA–protein interface blockers and programmable editors)

A complementary strategy is to block critical RNA–protein interfaces or retarget editing machinery rather than blunt enzyme catalysis globally. For the writer METTL16, aminothiazolones that disrupt the METTL16–MAT2A hp1 RNA interaction suppressed target engagement and provide a template for transcript-selective inhibition—potentially minimizing global on-target liabilities (145). In parallel, ADAR-recruiting editors (guide RNAs that enlist endogenous ADARs) have achieved increasingly precise *in vivo* A-to-I editing with wobble-enhanced designs, suggesting a route to therapeutic rescue of transcripts rather than enzyme inhibition per se (e.g., restoring antigen-presentation or apoptosis pathways). Although originally developed for genetic disease, these systems establish delivery and specificity principles directly relevant to oncology (146).

Consistency and gaps. Multiple groups now show that intercepting RNA–protein recognition (e.g., METTL16) or re-deploying ADAR catalysis can be done with drug-like or nucleic-acid scaffolds. The field still lacks oncology-focused, site-specific mRNA “anti-readers”/”anti-writers” with validated *in vivo* antitumor efficacy or scalable delivery (145).

### 4.5 Biomarker-guided combinations and delivery considerations

Convergent preclinical data support rational combinations. FTO inhibition + radiotherapy enhanced tumor control without worsening normal-tissue toxicity; reader targeting (YTHDF1/2) + RT or RT+ICB amplified STING–IFN-I signaling and T-cell priming; METTL3 inhibition combined with venetoclax overcame acquired resistance in AML. These pairings point to tractable, mechanism-matched regimens (152). On the delivery front, immune-cell-targeted nanoparticles (e.g., TAM/DC-directed carriers for YTHDF1 siRNA) and tumor-tropic ligands enable compartment-specific epitranscriptome editing while sparing non-target tissues—critical given widespread physiologic roles of RNA-modification enzymes (153). For patient selection, m^5^C/NSUN6–NDRG1 signatures predicted radioresistance in cervical cancer organoids/clinical cohorts, recommending pathway readouts as prospective biomarkers alongside m^6^A-DDR/ferroptosis panels (140) (**Table 2**).

**Table 2:** Biomarker panels and assays enabling patient selection for epitranscriptomic therapies.

Biomarker panel	Assay	Clinical utility
HR/m^6^A program activity	GLORI or m^6^A-SAC-seq; RAD51 foci; reader occupancy	Predict benefit from FTO/ALKBH5/METTL3 targeting; RT combos
Ferroptosis program	SLC7A11 m^6^A and protein; lipid peroxidation readouts	Guide ferroptosis inducers + writer/eraser targeting
Immune reader activity (DC/myeloid)	YTHDF1/2 protein; IFN-I induction post-RT; cathepsin translation	Flag readers as brakes on RT-elicited immunity; select for reader antagonism
m^5^C NSUN6-NDRG1 axis	RNA bisulfite-seq; ALYREF binding; NDRG1 levels	Stratify cervical cancer RT resistance
Editing burden & ADAR1	RNA editing index; ISG signatures	Select ADAR1 inhibitor + ICB/RT regimens

### 4.6 Safety, selectivity, and outstanding questions

Despite rapid progress, three challenges recur across studies. First, cell-type specificity: the same node (e.g., an m^6^A reader) can be anti-tumor in one compartment and pro-tumor in another; therefore, delivery restricted to malignant or defined immune lineages (TAMs, DCs) is likely to be essential (153). Second, durability and escape: whether tumors rewire to alternative readers (IGF2BP ↔ YTHDF families) or switch modification usage (e.g., ac^4^C/m^5^C compensation) under drug pressure remains untested over clinically relevant time scales (154). Third, on-target physiology: while STC-15 and FTO inhibition show promising therapeutic indices preclinically/early-clinically, systematic assessment of hematopoietic and neural side effects under chronic dosing is needed before broad combination trials (154).

Overall, the last five years have transformed RNA-modification proteins from intriguing biology into actionable drug targets. Catalytic inhibitors (METTL3, FTO, NAT10, emerging ADAR1 agents), protein degraders (METTL3/METTL14, FTO), reader antagonism (YTHDF1/2; IGF2BPs), and precision interface editing (METTL16–RNA disruption; ADAR recruitment) now constitute a diversified therapeutic toolbox (**Table 3**). The most compelling near-term paths pair these agents with radiotherapy, ICB, or standard cytotoxics, guided by assayable biomarkers that capture the active epitranscriptomic circuit in each tumor (144).

**Table 3:** Therapeutic strategies targeting RNA modification proteins.

Strategy	Example agents	Rationale	Likely partner therapy	Development stage
METTL3 inhibitor	STC-15 (oral), STM2457 (tool)	Writer inhibition to reduce DDR/niche retention	Chemo (AML), RT ± ICB (exploratory)	Phase 1 (STC-15)/Preclinical
FTO inhibitor	FB23-2, tool compounds	Impair HR; radiosensitize HNSCC	RT	Preclinical
ALKBH5 inhibitor	Emerging chemotypes	Lower DDR/ferroptosis tolerance	RT; cytotoxics	Preclinical
NAT10 inhibitor	Remodelin	Reduce ac^4^C stability of resistance transcripts	Dacarbazine; IO	Preclinical *in vivo*
ADAR1 inhibitor	Small molecules (preclinical)	Boost MDA5-IFN; reduce damage tolerance	ICB; RT	Preclinical
YTHDF1 antagonism/siRNA	Nanoparticles/EVs	Increase STING-IFN and cross-priming	RT; ICB	Preclinical
YTHDF2 antagonism	Genetic/pharmacologic (emerging)	Reprogram myeloid compartment	RT; anti-PD-L1	Preclinical
IGF2BP1 inhibitor	BTYNB & analogs	Block efflux/stemness mRNA stabilization	Cytotoxics	Preclinical
METTL16-RNA blocker	Aminothiazolones	Disrupt MAT2A hp1 recognition	Metabolic/chemo combos	Preclinical

## 5 Future research directions and prospects in tumor RNA modification research

A decade of work has made it clear that RNA modifications are not mere epiphenomena of stress but programmable control points for tumor fitness and antitumor immunity. The next phase should move from correlative catalogs to mechanism-anchored, patient-matched interventions. Below I outline priorities where recent primary data already provide credible launch pads—and where cross-group consistency or tension reveals what we still do not understand.

### 5.1 Quantitative, single-cell and spatial maps of the tumor epitranscriptome

Antibody-independent chemistries that report site identity and stoichiometry (e.g., GLORI and m^6^A-SAC-seq) now allow absolute, single-base quantification of m^6^A and should be brought into prospective oncology cohorts to track therapy-induced reprogramming *in situ*. These methods deliver stoichiometry rather than binary peak calls—exactly what is needed to relate modification “dose” to phenotype. Emerging algorithms for direct nanopore RNA signals (e.g., mAFiA) and single-cell protocols (picoMeRIP-seq; m^6^A-isoSC-seq) further enable isoform- and cell-state–resolved maps, a prerequisite for understanding heterogeneity within tumors and the TME. The field should prioritize paired pre/post-treatment biopsies and fractionated-RT sampling to capture early adaptive changes (155, 156).

### 5.2 Resolving reader specificity and redundancy—before we drug it broadly

Independent teams have reached different conclusions about how YTHDF paralogs partition function (translation *vs*. decay) and when they behave redundantly; similar ambiguities surround when YTHDFs versus IGF2BPs dominate mRNA stabilization. Mechanistic studies dissecting the low-complexity domains and condensate behavior argue for nonidentical roles of YTHDF1 and YTHDF2, while cancer models continue to implicate IGF2BPs in stabilizing drug-resistance transcripts (e.g., ABCB1). Systematic, cell-type–restricted perturbations in human tumors—ideally during therapy—are needed to adjudicate these models and to avoid off-target immunologic liabilities as reader inhibitors enter development (156).

### 5.3 Interfacing tumor-intrinsic DDR/ferroptosis with RT-elicited immunity

Two robust, orthogonal lines of evidence now show that m^6^A readers in the myeloid compartment (YTHDF1 in DCs; YTHDF2 in myeloid cells) act as brakes on the STING–IFN-I axis after irradiation, while tumor-intrinsic erasers (FTO; ALKBH5) and m^5^C writers (NSUN6) tune DNA repair and ferroptosis to set the radiation-response threshold. Future trials should test reader inhibition (or selective knockdown) as an immunologic adjuvant to RT and checkpoint blockade, while exploiting FTO/ALKBH5/NSUN6 inhibition to tilt DDR balance and cell death. Importantly, these strategies must be compartment-aware, as the same node can cut both ways in cancer versus immune cells (53, 152).

### 5.4 From tool compounds to patients: rational combinations and biomarkers

Clinical translation of METTL3 inhibition is underway (STC-15, phase I), and preclinical work supports combining writer or eraser inhibitors with standard therapies (e.g., venetoclax in AML; RT in HNSCC). The obvious next step is biomarker-guided trials that stratify by pathway activity—e.g., GLORI/m^6^A-SAC-seq–derived HR or ferroptosis signatures; NSUN6–m^5^C–NDRG1 readouts in cervical cancer; or reader-expression in tumor myeloid/DC compartments—to match patients to specific epitranscriptomic levers (138, 144).

### 5.5 Drugging the “hard” targets: readers and editors

Beyond catalytic writers/erasers, two drug classes are maturing. First, reader antagonism: IGF2BP1 inhibition with BTYNB induces leukemic differentiation *in vitro*, and new studies are mapping reader dependencies that could support medicinal chemistry campaigns; nanoparticle and EV platforms have already delivered anti-YTHDF1 siRNA *in vivo*, reshaping myeloid/TAM programs and enhancing therapy in liver cancer models. Second, ADAR1: multiple groups report bona fide small-molecule inhibitors (e.g., Rebecsinib; AVA-ADR-001) that amplify MDA5-dependent interferon signaling and show antitumor activity preclinically—opening the door to combination regimes with ICB and RT in ADAR1-high, immune-refractory tumors. Rigorous on-target selectivity, *in vivo* durability, and immune safety profiling will be critical as these programs mature (151, 153).

### 5.6 Precision epitranscriptome engineering and delivery

A parallel prospect is to modulate specific RNA–protein contacts or edits without globally altering the modification landscape—e.g., blocking METTL16–MAT2A hairpin recognition to throttle methionine/SAM-sensing, or recruiting endogenous ADARs to correct oncogenic edits. While oncology-focused demonstrations remain early, these approaches could minimize systemic toxicities. In the nearer term, lineage-targeted delivery (e.g., macrophage/DC-tropic nanoparticles) has proven feasible for siRNA cargoes and should be adapted to epitranscriptomic targets to respect the divergent roles of the same enzyme across compartments (83, 153).

### 5.7 Safety, resistance, and systems-level modeling

As first-in-human writer inhibitors and forthcoming reader/editor agents enter the clinic, three risks merit prospective study: (a) hematopoietic and neural liabilities of sustained m^6^A/ADAR modulation; (b) adaptive rewiring (e.g., shifting from YTHDF- to IGF2BP-dominated programs, or compensatory use of ac^4^C/*m*^5^C); and (c) emergent immune toxicities when re-activating STING/IFN circuits. Integration of multi-omic time series (modification stoichiometry, ribosome profiling, proteomics) with causal CRISPR maps—ideally under drug pressure—will help forecast and pre-empt escape routes; early genome-wide screens are already being used to uncover the genetic modifiers of METTL3 inhibitor sensitivity that can guide combination partners (63, 157).

### 5.8 A pragmatic path to the clinic

Convergently across disease sites, near-term opportunities include: pairing FTO or ALKBH5 inhibitors with RT to broaden the therapeutic window; testing reader antagonism (YTHDF1/2) to preserve RT-elicited STING–IFN and cross-priming, combining METTL3 inhibition with venetoclax or cytotoxics in AML; and deploying NAT10 inhibitors where ac^4^C programs drive resistance or immune suppression (melanoma, TNBC). Each of these now has primary *in vivo* support and tractable biomarkers for patient selection; the central challenge is *compartment-specific targeting* to maximize antitumor effects while sparing beneficial immune programs (152, 154).

The field is now at an inflection point: descriptive atlases have mapped the basic terrain, but the next step is to act with precision. What truly matters is not only detecting modifications, but asking *which mark, at which site, and in which cell type* meaningfully changes tumor–immune interactions. Therapeutic strategies will succeed only if they can match the right molecular target—whether a writer, reader, eraser, or editor—with the biological context, balancing tumor-intrinsic survival circuits against the immune responses that determine patient outcomes.
